# Using *In situ* Dynamic Cultures to Rapidly Biofabricate Fabric-Reinforced Composites of Chitosan/Bacterial Nanocellulose for Antibacterial Wound Dressings

**DOI:** 10.3389/fmicb.2016.00260

**Published:** 2016-03-04

**Authors:** Peng Zhang, Lin Chen, Qingsong Zhang, Feng F. Hong

**Affiliations:** ^1^Group of Microbiological Engineering and Industrial Biotechnology, College of Chemistry, Chemical Engineering and Biotechnology, Donghua UniversityShanghai, China; ^2^Key Laboratory of High Performance Fibers and Products, Ministry of Education, Donghua UniversityShanghai, China

**Keywords:** bacterial cellulose, chitosan, composite sheet, antibacterial wound dressing, horizontal rotating bioreactor, *in situ* dynamic culture technology

## Abstract

Bacterial nano-cellulose (BNC) is considered to possess incredible potential in biomedical applications due to its innate unrivaled nano-fibrillar structure and versatile properties. However, its use is largely restricted by inefficient production and by insufficient strength when it is in a highly swollen state. In this study, a fabric skeleton reinforced chitosan (CS)/BNC hydrogel with high mechanical reliability and antibacterial activity was fabricated by using an efficient dynamic culture that could reserve the nano-fibrillar structure. By adding CS in culture media to 0.25–0.75% (w/v) during bacterial cultivation, the CS/BNC composite hydrogel was biosynthesized *in situ* on a rotating drum composed of fabrics. With the proposed method, BNC biosynthesis became less sensitive to the adverse antibacterial effects of CS and the production time of the composite hydrogel with desirable thickness could be halved from 10 to 5 days as compared to the conventional static cultures. Although, its concentration was low in the medium, CS accounted for more than 38% of the CS/BNC dry weight. FE-SEM observation confirmed conservation of the nano-fibrillar networks and covering of CS on BNC. ATR-FTIR showed a decrease in the degree of intra-molecular hydrogen bonding and water absorption capacity was improved after compositing with CS. The fabric-reinforced CS/BNC composite exhibited bacteriostatic properties against *Escherichia coli* and *Staphylococcus aureus* and significantly improved mechanical properties as compared to the BNC sheets from static culture. In summary, the fabric-reinforced CS/BNC composite constitutes a desired candidate for advanced wound dressings. From another perspective, coating of BNC or CS/BNC could upgrade the conventional wound dressings made of cotton gauze to reduce pain during wound healing, especially for burn patients.

## Introduction

Bacterial nano-cellulose (BNC) is a natural cellulosic material, which is mainly secreted by acetic acid bacteria. The appearance of BNC heralds a green approach to obtain cellulose from the microbiological industry, a useful supplement to the agro-forestry industry (Klemm et al., 2005). BNC has more value other than a simple raw material because it is not just used as a new alternative cellulosic source, but also is an unparalleled functional material for its decent biocompatibility, nano-fibrillar supramolecular structure, hydrogel property, and super-high specific surface area, which is distinct from plant cellulose (Gatenholm and Klemm, 2010). Among them, the nano-fibrillar reticulate structure of microbially manufactured BNC is beyond what can currently be achieved artificially, and it looks like natural collagen in terms of its nanostructure and morphology, which is attractive for cell immobilization and because it mimics extracellular matrix (ECM) support (Petersen and Gatenholm, 2011). The current popular approach for fabrication of BNC composites destroys the innate nano-fibrous microstructure of BNC, which would impair its functionality that is based on its native morphology. Thus, conservation of BNC's natural nano 3D network structure and its corresponding features is an important consideration in the development of different functional BNC composites. Owing to BNC's unique bacterial cultivation procedure, a methodology of *in situ* static bio-synthesis is commonly applied to produce BNC composites (Phisalaphong and Jatupaiboon, 2008; Wesarg et al., 2012). Specifically, in the *in situ* bio-synthesis process, the composites are generated through adding various materials into culture media during or before cultivation instead of using any post-processing to alter an existing BNC hydrogel, for instance, to make other substances integrated into the BNC hydrogel matrix. As previously reported, the added materials may include soluble polymers (Phisalaphong and Jatupaiboon, 2008), particles (Yan et al., 2008), fibers (Pommet et al., 2008), and even some block fabrics (Meftahi et al., 2010).

Recently, BNC has been reported to possess a natural and intrinsic power to heal wounds (Czaja et al., 2006). Diverse BNC-based wound dressings, skin substitutes, and skin tissue repair materials have been developed to treat different traumas, such as burns (Fontana et al., 1990; Czaja et al., 2007), chronic wounds (Serafica et al., 2010), and full thickness skin injury (Fu et al., 2012). BNC is also favored in the development of various implanted medical devices, which include artificial menisci (Bäckdahl et al., 2006), vascular grafts (Bäckdahl et al., 2011; Hong et al., 2015; Tang et al., 2015), articular cartilages (Lopes et al., 2011; Martínez Ávila et al., 2014), and scaffolds for tissue regeneration (Bodin et al., 2010; Zaborowska et al., 2010). To satisfy miscellaneous applications, BNC has to be modified to improve its mechanical properties (Nakayama et al., 2004; Brown et al., 2011), rehydration abilities (Lin et al., 2009; Chen et al., 2011), antibacterial activity (Phisalaphong and Jatupaiboon, 2008), biocompatibility (Ciechańska, 2004; Cai and Kim, 2010), and diverse biological functions (Phisalaphong and Jatupaiboon, 2008). Among the properties, the mechanical strength to endure stress in actual use and the antibacterial activity to reduce infection risks are two general demands for most biomedical applications.

Although, the high number of intramolecular and intermolecular hydrogen bonds make the tensile strength of BNC much higher than many other natural materials, BNC itself is still unable to completely meet the requirements of wet wound dressings (Clasen et al., 2006) or the demands of other applications that need excellent mechanical performance (Nakayama et al., 2004). The mechanical properties of BNC hydrogel can be strengthened through reducing the porosity or lowing water content (Clasen et al., 2006; Retegi et al., 2010), compositing with other polymers to form a double network (Nakayama et al., 2004; Hagiwara et al., 2010), or cross-linking via glyoxalization (Quero et al., 2011), all of which certainly alter the inherent structure or chemical composition of BNC hydrogel. Reinforcing hydrogel with a fabric skeleton, by contrast, is a desirable approach to significantly enhance the mechanical properties without dramatically altering the native features of hydrogels (Agrawal et al., 2013). For instance, involvement of fabrics remarkably improved tear resistance of poly(2-hydroxyethyl methacrylate; pHEMA) hydrogel for artificial skin use (Young et al., 1998). Since, conventional hydrogels are formed from monomer solutions or dispersive polymer suspensions, it is easy to embed a frame network into gel matrix through immersing fabrics in raw polymer solutions or monomer solutions followed by cross-linking or polymerization. An analogous approach used for embedding fabric skeletons into BNC hydrogel is to place the fabrics in culture media directly. However, under current static culture conditions, the fabrics as skeletons must float on the surface of culture media since BNC is only excreted to near the air-liquid interface (<1 mm in depth; Verschuren et al., 2000; Hornung et al., 2006), which leads to the fabric being half-encapsulated and half-unmodified in one culture cycle (Meftahi et al., 2010).

Chitosan (CS), a natural amino polysaccharide, possesses many outstanding properties, such as the biodegradability, biocompatibility, non-toxicity, healing promotion, and especially antibacterial properties. Therefore, it is highly promising to combine it with BNC to develop ideal functional composites for biomedical applications. The CS/BNC composites have been fabricated in various ways, such as impregnating the purified BNC matrix in CS solution (Kim et al., 2011; Ul-Islam et al., 2011; Lin et al., 2013), casting of CS-blended BNC homogenate suspension (Fernandes et al., 2009; Nge et al., 2010) or casting of CS-blended BNC co-solution (Wu et al., 2004), and *in situ* static bio-synthesis (Ciechańska, 2004; Phisalaphong and Jatupaiboon, 2008). Most results showed CS/BNC composites gained antibacterial effects and improved other properties including moisture retention, and bioactivity. By using the impregnation method, CS penetrates completely into BNC matrix via free and physical diffusion, which needs a prolonged period for soaking and depends on molecular weight and concentration of CS, as well as pore sizes of BNC network. The padded CS in the original interstices of BNC would possibly be lost by subsequent diffusion in practical applications. In the casting approach, the inherent microstructures of BNC hydrogel will be entirely disrupted. Therefore, the *in situ* biosynthesis method, where chitosan is supplied in bacterial cultures, is supposed to be better to develop CS/BNC composites. However, the antimicrobial property of chitosan may inhibit the bacterial growth and decrease cellulose production, resulting in very thin CS/BNC sheets (Ciechańska, 2004). Besides, the crude BNC composites obtained by *in situ* biosynthesis have to be subjected to a routine purification process composed of a series of alkaline boiling and water washing steps to achieve a medical grade non-pyrogenic material before clinical uses (McKenna et al., 2009; Martínez Ávila et al., 2014). During the purification process, the loosely bound CS could be completely removed from the BNC matrix (Heßler and Klemm, 2009).

In order to solve the problems with preparation of the CS/BNC composites in static cultures, dynamic cultures were therefore proposed to produce fabric-embedded CS/BNC hydrogel sheets in a horizontal rotating bioreactor. In this approach, cotton gauze used as a mode fabric was fixed to form a rotating drum to immobilize CS/BNC hydrogel as the support during bacterial cultivation. By compositing, the embedded fabric functioned as the inner skeleton of the composite to reinforce the hydrogel. This process was expected to minimize the negative antibacterial impacts of chitosan during cultivation. *In situ* dynamic biosynthesis of CS/BNC composite sheets was performed in the presence of three CS addition concentrations (0.25, 0.50, and 0.75%, w/v) and was compared with the conventional CS/BNC composites from static cultures. The characteristics including tensile properties, water holding and absorption properties, and antibacterial properties of the fabric-reinforced CS/BNC composites were investigated in detail.

## Materials and methods

### Microorganism

The bacterial strain used in this study was *Gluconacetobacter xylinus* ATCC 23770 (obtained from American Type Culture Collection, Manassas, VA), which was preserved on agar slant at 4°C. One loop of colonies was inoculated into a culture medium containing 2.5% (w/v) D-mannitol, 0.5% (w/v) yeast extracts, 0.3% (w/v) peptone with an acetic acid-adjusted pH of 5.0, and then cultivated with a shaking speed of 160 rpm at 30°C for 12 h to prepare seed culture.

### Preparation of fabric-reinforced CS/BNC composites

#### Preparation of culture medium

The chitosan with viscosity of 50–800 mPa·s (average MW = 620 kDa) and degree of deacetylation 92% (Sinopharm Chemical Reagent Co., Ltd, Shanghai, China) was dissolved in diluted acetic acid to reach a concentration (w/v) of 0.25, 0.5, and 0.75% separately, and then was premixed with 2.5% (w/v) glucose, 0.5% (w/v) yeast extract, 0.3% (w/v) peptone. Higher concentration of chitosan-added medium was not achieved because of co-dissolving difficulties with the culture substrates. The chitosan-free medium with the same formula was used as the control. The pH value in all the culture media was adjusted to 4.5 with acetic acid before autoclaved at 121°C for 20 min.

#### Design of the horizontal rotating bioreactor

**Figure 1:**
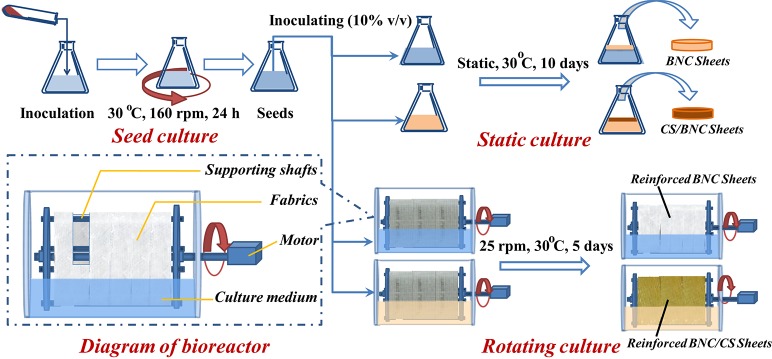
**Preparation processes of the CS/BNC composites with rotating culture and static culture**.

A laboratory-scale horizontal rotating bioreactor was constructed and applied in this study. As shown in Figure 1, the bioreactor is equipped with a horizontal glass cylinder as its container and a rotating roller cage (9.0 cm in diameter) used to fasten fabric (Hong et al., 2012a). The roller cage is rotated via a central spindle connected to a controllable motor. The top half of glass cylinder could be opened to remove or replace the roller cage. The dry weight of cotton gauzes was pre-weighed before installation onto roller cage for cultivation. The fabric-equipped bioreactor was autoclaved without media at 121°C for 20 min and then placed in a sterile safety cabinet before inoculation.

#### Preparation of CS/BNC composites

The fabric-reinforced CS/BNC composites in rotating cultures (Hong, 2012b) and the CS/BNC composite sheets in static cultures were prepared, respectively, following the procedures as shown in Figure 1. Rotating cultivation was performed by transferring 400 mL inoculated culture medium into the horizontal bioreactor with 10% (v/v) inocula and then cultivated with an optimized rotating speed of 25 rpm at 30°C for 5 days. Four parallel cultures were carried out for each CS concentration of 0, 0.25, 0.5, and 0.75%. After cultivation, the fabric-reinforced CS/BNC composite sheets were harvested from the roller. At the same time, a static cultivation in quadruplicate was carried out in 250 mL flasks containing 100 mL inoculated media for 10 days as the control. The number of viable bacterial cells in the liquid media was monitored through serial dilutions and spread plating.

#### Purification and gravimetric analysis of CS/BNC composites

A routine harsh purification procedure (McKenna et al., 2009) was employed in this research, including incubation in 0.5% (w/v) NaOH aqueous solution (renewed for each 2 h) at 80°C for 8 h to remove bacterial cells and other ingredients, followed by rinsing with deionized water until pH was neutral.

The purified samples were completely dried at 105°C to a constant weight for gravimetrical measurements. The total dry weight of BNC or CS/BNC coat of the composites from rotating cultivations was obtained by subtracting the dry weight of cotton gauze from the total weight of cotton gauze-reinforced BNC or CS/BNC composites. The total dry mass of BNC or CS/BNC hydrogels from static cultivations was directly obtained by gravimetrical measurements.

### Characterization of CS/BNC composites

#### Microstructure inspection

The reinforced CS/BNC and the pristine BNC were freeze-dried in swollen state and were sprayed with gold for observation. The microstructure of the samples was observed with a field emission scanning electron microscope (FE-SEM, S-4800 model, Hitachi, Japan).

#### Thermogravimetric analysis

The CS/BNC coat layer was stripped with a lancet from a reinforced CS/BNC composite sheet after freeze-dried. The CS/BNC coat sample of 3–5 mg was weighed and loaded into an alumina oxide pan to carry out the thermal gravimetric analysis (TGA) by using a Netzsch TG-209-F1 Libra gravimetric analyzer (Netzsch, Germany). The samples were heated from 25 to 900°C with a heating rate of 10 K min^−1^ under the protection of nitrogen flow (250 mL min^−1^).

#### Content of chitosan

The content of chitosan in the CS/BNC composite coat (excluding fabric skeleton) was obtained using the elemental analysis method. Nitrogen percentage of CS/BNC composite (N_CS∕BNC_), of chitosan (N_CS_), and of BNC (N_BNC_) was detected with an elemental analyzer (Vario EL III, Elementar Analyser Systems GmbH, Hanau, Germany). Both total mass and total nitrogen of CS/BNC composite are supposed to be assigned to CS portion and BNC portion:

(1) $$\begin{array}{r} \text{Total }{\text{m}}_{\text{CS}∕\text{BNC}}={\text{m}}_{\text{CS}}+{\text{m}}_{\text{BNC}} \end{array}$$

(2) $$\begin{array}{r} \text{Total }{\text{N}}_{\text{CS}∕\text{BNC}}={\text{P}}_{\text{CS}}+{\text{P}}_{\text{BNC}} \end{array}$$

where, Total m_CS∕BNC_, m_CS_, and m_BNC_ is the total mass of CS/BNC composite, the mass of CS and BNC component, respectively. Total N_CS∕BNC_ is the total nitrogen in CS/BNC composite that is composed of CS portion (P_CS_) and BNC portion (P_BNC_, although very little). Thus, the element conservation Equation (2) of nitrogen can be specified as:

(3) $$\begin{array}{l} \begin{array}{c} \text{Total }{\text{N}}_{\text{CS}∕\text{BNC}}={\text{N}}_{\text{CS}∕\text{BNC}}\times {\text{m}}_{\text{CS}∕\text{BNC}}={\text{P}}_{\text{CS}}+{\text{P}}_{\text{BNC}}\\ ={\text{N}}_{\text{CS}}\times {\text{m}}_{\text{CS}}+{\text{N}}_{\text{BNC}}\times {\text{m}}_{\text{BNC}}\\ ={\text{N}}_{\text{CS}}\times {\text{m}}_{\text{CS}}+{\text{N}}_{\text{BNC}}\times \left({\text{m}}_{\text{CS}∕\text{BNC}}-{\text{m}}_{\text{CS}}\right) \end{array} \end{array}$$

CS content (α_cs_) in the CS/BNC composite was calculated with the following rearranged equation:

(4) $$\begin{array}{l} \text{CS content}\left(\alpha_{\text{CS}}\right)=\frac{{\text{m}}_{\text{CS}}}{{\text{m}}_{\text{CS}∕\text{BNC}}}=\frac{{\text{N}}_{\text{CS}∕\text{BNC}}-{\text{N}}_{\text{BNC}}}{{\text{N}}_{\text{CS}}-{\text{N}}_{\text{BNC}}}\times 100\% \end{array}$$

#### ATR/FT-IR

Molecular structure variations of CS/BNCs were investigated with an infrared spectrophotometer equipped with attenuated total reflectance (FTIR-8400, Shimadzu Co. Japan) and the spectra from 4500 to 600 cm^−1^ were recorded.

#### Water holding and absorption characteristics

The water absorption capacity was measured according to the British Pharmacopeia monograph for alginate dressings and packing. The hydrogel samples were cut to rectangles of 2 × 4 cm sizes and were weighed as initial weight (W_0_), after that the samples were freeze-dried and then were soaked in solution A (containing 2.5 mmol/L CaCl_2_·2H_2_O and 142 mmol/L NaCl which simulate the ionic strength of Ca^2+^ and Na^+^ in wound exudates) for 30 min. The wet samples were weighed (W_1_) after picked out and hung up with a forceps for 30 s to remove the surface liquid. After that, the samples were transferred to a centrifuge tube whose bottom was padded knitted gauze to absorb squeezed water. The samples were centrifugally dehydrated for 15 min at 1200 rpm and were weighted (W_2_). The ultimate weight of completely dried samples (W_3_) was weighed after drying at 105°C. The water holding capacity (WHC) and water absorption capacity (WAC) was calculated as:

(5) $$\begin{array}{r} \text{WHC}=\frac{{\text{W}}_{0}-{\text{W}}_{3}}{{\text{W}}_{3}} \end{array}$$

(6) $$\begin{array}{r} \text{WAC}=\frac{{\text{W}}_{1}-{\text{W}}_{3}}{{\text{W}}_{3}} \end{array}$$

Fluid held within the sample was divided into two parts: fluid held between fibers (W_1_ − W_2_) and fluid held inside the individual fibers (W_2_ − W_3_). And the normalized parameters (W_1_ − W_2_)/W_3_ and (W_2_ − W_3_)/W_3_, which expressed the fluid intake of per gram sample (g/g), were used to compare dressing materials (Qin, 2004). The ratio of (W_1_ − W_2_)/(W_2_ − W_3_) was also evaluated, indicating distribution of fluid in dressing (Qin, 2004, 2008). Each test was repeated eight times, and then mean values ± standard deviations were given.

#### Antibacterial performance tests

**Figure 2:**
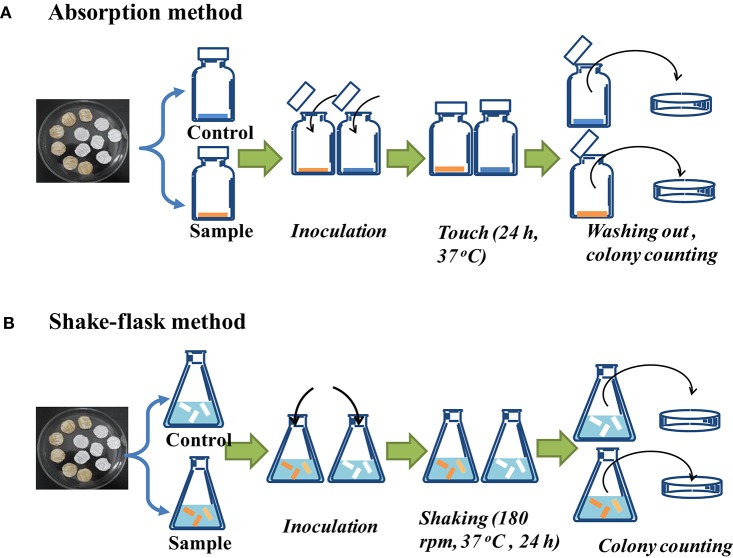
**Protocol of the antibacterial ability test: (A) absorption method (ISO 20743-2007), (B) shake-flask method (saline)**.

Considering both wet and dry BNC materials (i.e., hydrogel and dehydrated BNC) have the respective advantages in therapy of chronic traumas and burns (Fu et al., 2013), and treatment of acute traumas (Wei et al., 2011), the antibacterial performances of the fabric-reinforced samples both in hydrogel state and lyophilized state against *Escherichia coli* and *Staphylococcus aureus* were evaluated. Two quantitative evaluation methods, the absorption method (Figure 2A) and the shake flask method (saline, Figure 2B) were applied. In the former method, the bacterial suspension immediately touched with the samples by liquid absorption, while in the shake flask method the composite samples were cultured in a dynamic germy liquid culture medium by shaking. A gauze-reinforced BNC sample was used as control. The test procedures are described as follows:

(A). Absorption method (ISO 20743-2007)

In the absorption method (Figure 2A), the bacterial inoculum directly contacted with control and test samples by pipetting inocula on the surface of samples. As shown in Figure 2A, the absorption method was performed according to ISO20743-2007 standard. An adjusted germy inoculum (1 × 10^5^ to 3 × 10^5^ cfu/mL) of 0.2 mL was scattered on the sample surface in a vial and tighten the cap. After touching with the sample for 24 h, 20 mL Soya Casein Digest Lecithin Polysorbate (SCDLP) base broth medium, consisting of 17 g/L peptone from casein, 3 g/L soy peptone, 5 g/L NaCl, 2.5 g/L KH_2_PO_3_, 2.5 g/L glucose, 1 g/L lecithin, and 7 g/L polysorbate 80, was added into the vial and washed out the cells in the samples using a vortex mixer. The number of viable cells was quantified by spread plating of serial dilutions. The gauze-reinforced BNC samples in hydrogel and lyophilized states were used as controls and each sample had three parallels. The percentage reduction (R), growth value of control (F), and antibacterial activity value of test (A) were calculated as follows:

(7) $$\begin{array}{rcl} \text{R}\left(\%\right) & = & \frac{{\text{C}}_{\text{t}}-{\text{T}}_{\text{t}}}{{\text{C}}_{\text{t}}}\times 100\% \end{array}$$

(8) $$\begin{array}{rcl} \text{F} & = & \mathrm{lg}{\text{C}}_{\text{t}}-\mathrm{lg}{\text{C}}_{0} \end{array}$$

(9) $$\begin{array}{rcl} \text{A} & = & \mathrm{lg}{\text{T}}_{\text{t}}-\mathrm{lg}{\text{C}}_{\text{t}} \end{array}$$

where, C_t_, T_t_, and C_0_ was the average concentration of bacterial cells of control sample at 24 h, of test sample at 24 h, and of control sample at 0 h, respectively.

(B). Shake-flask method (saline)

As shown in Figure 2B, the shake-flask method (saline) was performed according to Singh's method (Singh et al., 2012). The sample of 0.75 g was cut into small pieces, and then soaked in a flask containing 70 mL PBS and 5 mL germy medium (3 × 10^5^ to 3 × 10^6^ cfu/mL). Then the flask was cultivated with a shaking speed of 180 rpm at 37°C for *E. coli* and *S. aureus*. The number of viable cells in the shake-flask after inoculation (0 h) and shaking for 24 h was detected using spread plating of serial dilutions. The gauze-reinforced BNC samples in hydrogel and lyophilized states were used as controls and each test was performed in three parallels. The percentage reduction (R), growth value of control (F), and antibacterial activity value of test (A) were calculated as the same as that in the absorption method.

#### Mechanical properties

The water-saturated samples were cut into rectangles of 1 × 4 cm. The tensile evaluation was performed on a universal material testing machine (H5K-S, Hounsfield Test Equipment Ltd., England) under ambient condition (25°C, around 50% relative humidity) at a speed of 50 mm/min with a 100 N sensor loaded. Each test was repeated eight times, and then mean values ± standard deviation were given.

#### Statistic

In the analysis of the mechanical and antibacterial properties, the statistical significance was tested by one-way analysis of variance (ANOVA). A *post-hoc* test (Tukey HSD) was applied to specify differences among means. *P* < 0.05 was considered as significant.

## Results and discussion

### Bio-fabrication of fabric-reinforced BNC and CS/BNC composites

**Figure 3:**
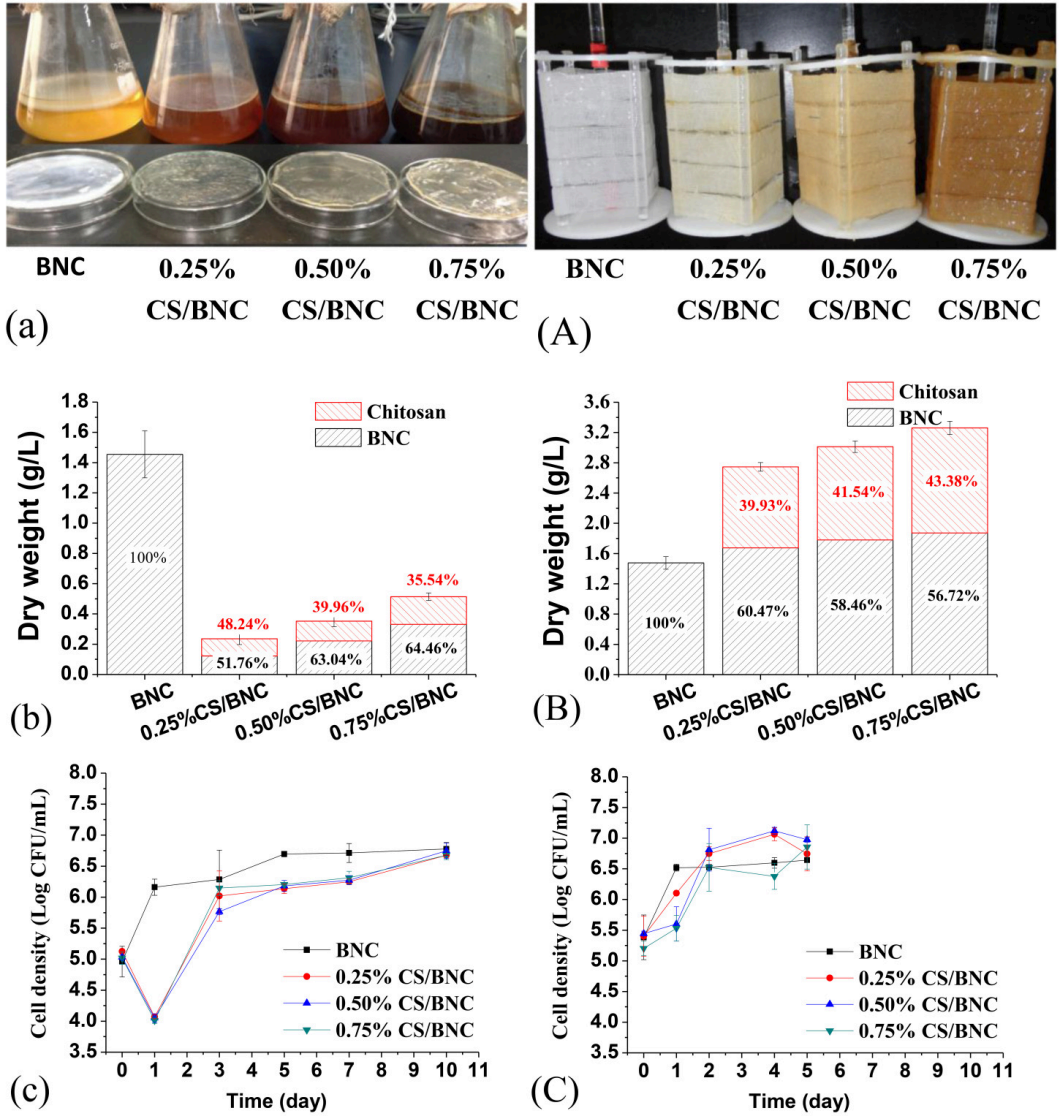
**Biosynthesis of the CS/BNC composites: appearance (after purification) of BNC and CS/BNC composites in static cultures (a) and fabric-reinforced BNC and CS/BNC composites in rotating cultures (A)**. The dried weight of BNC, CS/BNC sheets and composite components in static cultures and rotating cultures are show in **(b,B)**, respectively. The cell growth curves in static cultures **(c)** and rotating cultures **(C)** are shown as well. The experiments were performed in triplicate, and standard deviations were given as error bars.

CS/BNC composite sheets were bio-fabricated *in situ* by using both static culture and rotating culture (as shown in Figure 1). Chitosan solution is a usually transparent and pale yellow liquid but turns brown after autoclaving sterilization due to Maillard reactions between -NH_2_ and -OH groups (Yang et al., 2007), which could bring about an increasing color intensity in both the culture broth and the resulting hydrogel sheets (as shown in Figures 3a,b). In the static culture, the composite sheets floated on the surface of culture broth, while in the rotating culture the fabric-reinforced composite sheets formed via rotation coating and *in situ* biosynthesis of BNC on the fabric skeleton. In the former situation, the production efficiency of pristine BNC was far lower than that in the rotating culture, which should be ascribed to defects in mass transfer (Hornung et al., 2006). Therefore, a longer cultivation period of 10 d was required for static culture to obtain the thickness of pristine BNC sheet of 2.50 ± 0.35 mm, while only 5 d rotating cultivation was needed for the fabric-reinforced BNC sheet to achieve similar thickness (2.49 ± 0.32 mm). With addition of chitosan to culture medium to low concentration (0.25–0.75%, w/v), the production of hydrogel sheets in the static culture was strongly inhibited and sheet thickness decreased drastically (Figure 3a). CS/BNC composite sheets produced in static culture had a regular appearance on the air interface side, but the underside of the sheets adhered only as a loose gel that was weak and formless. This gel could be completely removed in a subsequent alkaline purification treatment. The thicknesses of iCS/BNC (*i* was 0.25, 0.50, 0.75%, respectively) sheets obtained from the static cultures were all less than 0.10 mm after purification, and their volume dry weights were 0.24, 0.35, 0.51 g/L for 0.25%CS/BNC, 0.50%CS/BNC, and 0.75%CS/BNC, respectively. By element analysis (EA), the detected nitrogen percentage in the pristine BNC was lower than 0.12% (w/w), indicating residues of cells and culture medium in the BNC matrix were little after the alkaline purification. Thus, the deductive CS content in the dry CS/BNC composite coat (excluding fabric) based on nitrogen percentage was determined as 48.24, 39.96, and 35.54% (w/w) for 0.25%CS/BNC, 0.50%CS/BNC, and 0.75%CS/BNC, respectively, which was 0.12, 0.22, and 0.33 g/L corresponding to the absolute yield of composite (Figure 3b). Surprisingly, the absolute yield of composite rose slightly with the increase of chitosan addition from 0.25 to 0.75% (w/v). One possible reason for the increase could be the addition of acetic acid that was used for dissolving chitosan, which could be utilized as a carbon source for *G. xylinus*. Previous studies have shown that acetic acid can enhance BNC yield (Yang et al., 2014). However, the cellulose production was much lower after adding chitosan than that in the chitosan-free culture medium (1.46 g/L), indicating a serious inhibiting effect on cellulose production under static culture condition. Figure 3c also shows that growth of *G. xylinus* was inhibited since the cell density decreased in the initial 24 h after inoculation, but could rebound in the next 48 h, indicating an adaptiveness of *G. xylinus* against low concentration of CS. Since the CS/BNC composite sheets obtained from static cultures were too fragile and thin to accomplish thorough evaluations (like mechanical tests or antibacterial tests), it is less possible for practical use and therefore was not evaluated further in this study.

By utilizing fabrics as the support in a self-designed horizontal rotating bioreactor (Hong et al., 2012a; Zhang et al., 2013), BNC or CS/BNC hydrogel sheets were satisfactorily developed through *in situ* biosynthesis on the fabrics. The appearance of fabric-reinforced CS/BNC and -reinforced BNC composites after purification was shown in Figure 3A. Unlike the CS/BNC sheets obtained in static cultures, dynamically-cultured CS/BNC hydrogel sheets showed compact, stable, and symmetrical appearances. The CS/BNC sheets had a fairly good thickness of 2.68 ± 0.42, 2.69 ± 0.33, and 2.84 ± 0.45 mm for 0.25%CS/BNC, 0.50%CS/BNC, and 0.75%CS/BNC, respectively, which was slightly higher than the reinforced pristine BNC sheets (2.49 ± 0.32 mm). The fabric skeletons could be seen through the transparent hydrogel coat and the color of the iCS/BNC hydrogel coat changed from completely colorless transparency of pristine BNC to tawny with the increase of CS addition from 0.25 to 0.75%. The coating amount of hydrogel was 30.75, 56.41, 61.81, and 66.91 g/m^2^, corresponding to the absolute output of 1.48, 2.71, 2.97, and 3.21 g/L (without the weight of fabrics), for the additive concentrations of chitosan of 0, 0.25, 0.50, and 0.75%, respectively. The CS content in the CS/BNC composite coat obtained with the CS addition of 0.25, 0.50, and 0.75% (w/v) was 39.52, 41.53, and 43.27%, respectively. The only cellulose output in the 0.25–0.75% CS-added medium was 1.63, 1.73, and 1.82 g/L, respectively, which increased with CS addition and was slightly higher than the CS-free medium (1.48 g/L), as shown in Figure 3B. The results indicate that CS could be incorporated into the BNC successfully when it was produced in the rotating culture system, and the CS could be retained in the BNC matrix stably even after undergoing a harsh alkaline purification. Compared with the *in situ* static culture, the inhibiting effects on cellulose production were eliminated in the rotating culture. The cell growth curves in the rotating culture (Figure 3C) did not show the remarkable reduction that was found within 1 d in the static culture, although the growth rate in the CS-added culture was slower than the CS-free culture in the initial 24 h after inoculation. This result indicates that the present CS addition gave a low level inhibition effect on bacterial growth in the rotating culture.

CS is a natural antibacterial polymer, which can disrupt cytomembrane structure, cellular energy metabolism, and protein synthesis (Raafat et al., 2008; Galván Márquez et al., 2013). As CS is a dose-dependent bactericide (Raafat et al., 2008), addition of a small amount of CS into culture medium would not completely inhibit the cell proliferation and cellulose production, even could make CS incorporate into BNC matrix successfully during *in situ* biosynthesis. Static culture appeared to be more susceptible than rotating culture to the addition of CS. Under the negative influence of CS, only very thin BNC composite sheets (less than a quarter of BNC mass in the CS-free culture, Figure 3b) could be harvested in the static cultures as reported in literature (Ciechańska, 2004), while the same effects were not observed in the rotating cultures (Figure 3B). One possible explanation is that rotating cultures provide a better nutrient and oxygen transport than static cultures, which is conducive to bacterial proliferation and metabolism (Serafica et al., 2002) and consequently enhances bacterial adverse-resistant activity. The other explanation is that the immobilization of BNC hydrogel on the fabric support is a natural form of cell immobilization, just like biofilm, which helps *G. xylinus* cells to achieve higher regional cell numbers and to resist the harmful circumstances (Cheng et al., 2009; Maksimova, 2014). Thus, compared with the static cultures, the bacteria were able to grow faster in the rotating cultures to achieve a higher cell density and to get a quick adaption to the CS-added cultures, which can be concluded from the growth curves (Figures 3c,C).

### Characterization of fabric-reinforced BNC and CS/BNC composites

#### Morphology

**Figure 4:**
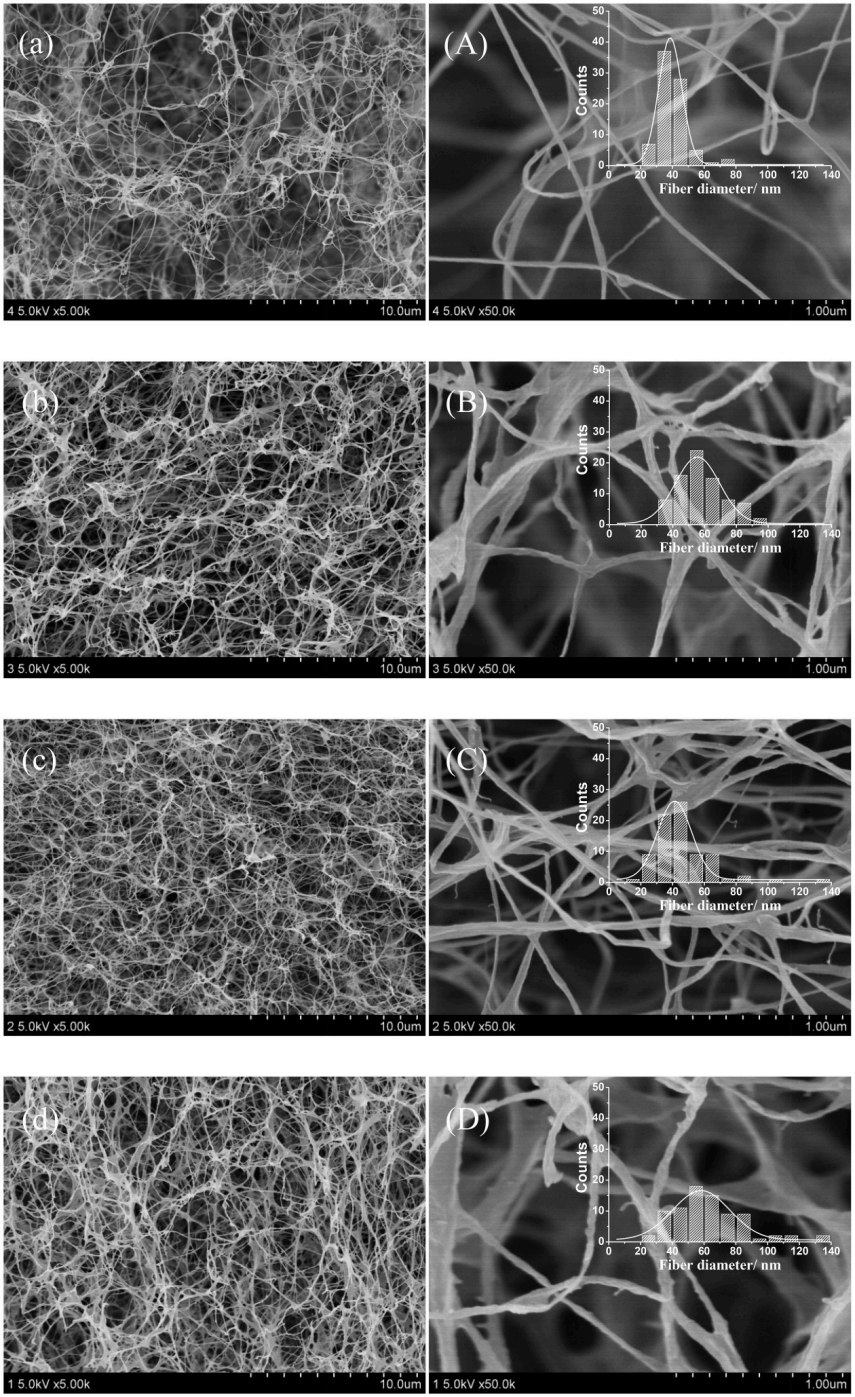
**FE-SEM micrographs of the hydrogel coat layer of the reinforced BNC (a,A), the reinforced 0.25%CS/BNC (b,B), the reinforced 0.50%CS/BNC (c,C), and the reinforced 0.75%CS/BNC (d,D) at × 5000 and × 50,000 magnification, respectively**. Embedded small graphs show the diameter distribution of the nanofibers.

Since it is difficult to prepare the fracture surface of the whole fabric-reinforced BNC composites for SEM inspection, the cross section of the hydrogel coat layer striped from the fabric-reinforced BNC and CS/BNC composites was examined with FE-SEM, as shown in Figure 4. Figures 4a,A show a typical nano-fibrous network of BNC, whose fiber diameters mostly range in a narrow scope between 20 and 60 nm with an average value of 39.1 nm. With addition of 0.25, 0.50, and 0.75% (w/v) CS, the average size of single fiber increased to 54.9, 41.8, and 58.8 nm, respectively. And the distribution of fiber diameters tends to turn much wider (Figures 4B–D). Distinct morphological difference between BNC and CS/BNC was that the BNC fiber was fairly uniform, while CS/BNC fiber size was irregular even for single fiber. This may result from CS disturbing the normal aggregation of BNC fibrils as it incorporates into the BNC fibers. Besides distribution of fiber diameter, CS/BNC network was much denser than BNC. In contrast, CS/BNC composites prepared by soaking BNC sheets in a CS solution have massive visible depositions of CS that fill in empty spaces of fibrillar network of BNC or coat on the surface of BNC fibers (Kim et al., 2011; Ul-Islam et al., 2011), which dramatically alters the original morphology to a greater degree than the CS/BNC composites from the *in situ* rotating cultures. The FE-SEM results showed that CS was perfectly dispersed and composited with internal nano-fibers of BNC. Overall, hydrogel coat of the CS/BNC composites obtained in the rotating cultures remained a similar nano-fibrillar structure as pristine BNC, and more importantly, multiple unique structure-related features of BNC could also be remained (i.e., continuous porosity, ECM-likeness, large surface areas, etc.).

#### IR and TGA analysis

**Figure 5:**
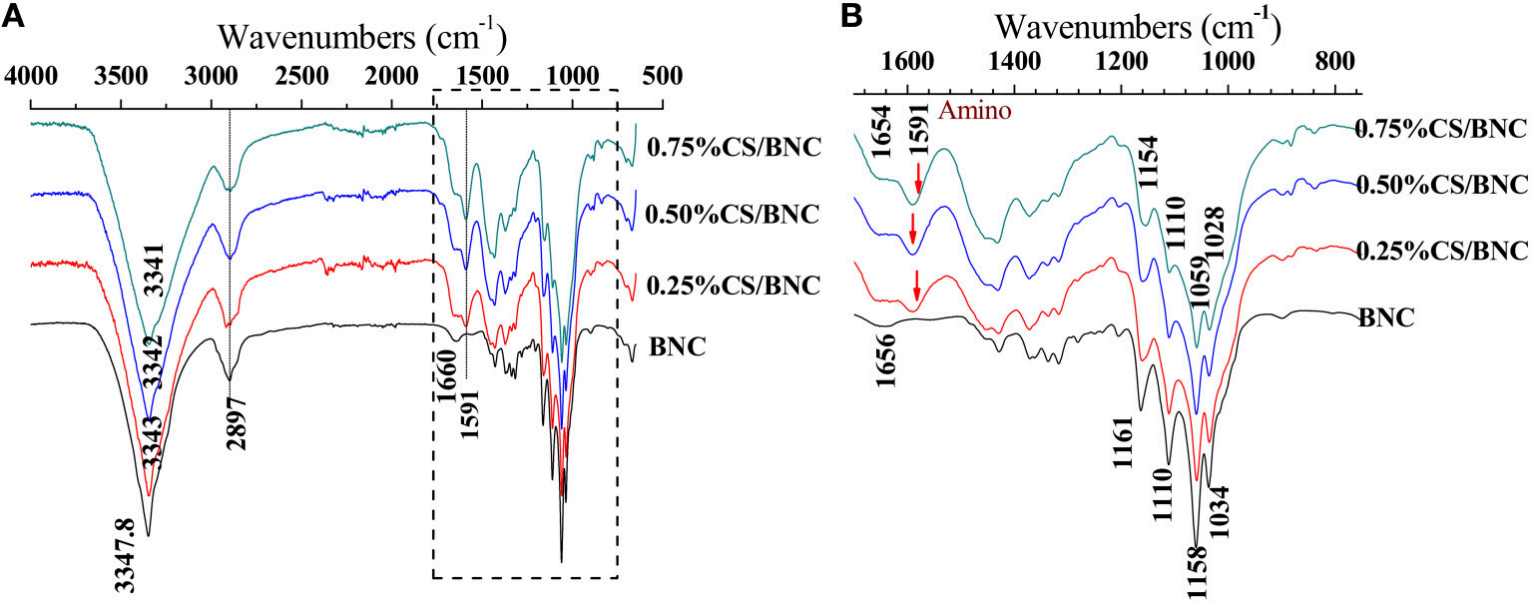
**ATR-IR spectra of fabric-reinforced BNC and fabric-reinforced CS/BNC composites: (A) spectra at wavenumber ranging from 4000 to 500 cm^**−1**^, (B) spectra are amplified at wavenumber ranging from 1700 to 800 cm^**−1**^**.

ATR-IR spectra of pristine BNC and CS/BNC composites are shown in Figure 5. Chitosan and cellulose both possess similar chemical structure of repeating D-glucopyranose unites linked with β-1,4 glycosidic bonds. In the case of pure BNC coat peeled from the fabric-reinforced composite, a sharp characteristic peak of O-H stretching appears at 3347.8 cm^−1^ while the C-H stretching appears at 2897 cm^−1^. Another important characteristic peak of 1660 cm^−1^ is assigned to the glucose carbonyl stretching (Kim et al., 2011). A series of bands range from 1200 to 1000 cm^−1^ fingerprint, which are related to the stretching of C-O-C of sugar rings and C-O stretching vibrations of the primary (C6) and secondary hydroxyl (C2, C3). For CS/BNC composites, an additional sharp absorption at 1591 cm^−1^ for N-H bending vibrations is distinguished from the cellulose bands, illustrating the CS has been successfully composited with BNC and can retain in the BNC matrix after alkaline purification. And the intensity of peak increases along with the additive concentration rising from 0.25 to 0.75% (w/v). Compared to the O-H stretching peak of BNC at 3347.8 cm^−1^, the maximum absorption band of CS/BNC composites shifts to lower wave numbers (3443.0, 3342.3, 3341.0 cm^−1^ for 0.25%CS/BNC, 0.50%CS/BNC, and 0.75%CS/BNC respectively), implying a decreased degree of hydrogen bonding (Clasen et al., 2006).

**Figure 6:**
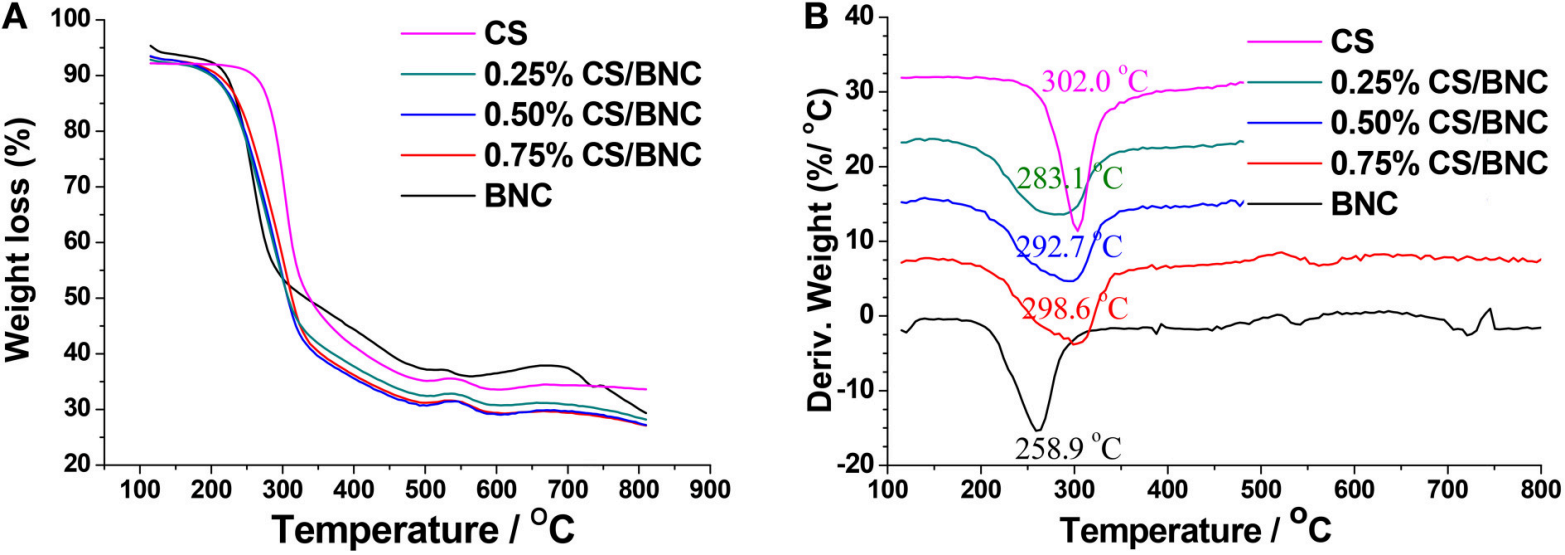
**Typical thermal degradation profiles (A) and derivative curves (B) for the hydrogel coat of fabric-reinforced BNC and -reinforced CS/BNC composites**.

Thermo-gravimetric analysis (TGA) was performed with only the hydrogel coat parts of the fabric-reinforced BNC and -reinforced CS/BNC composites. Figure 6A shows the weight loss of BNC, CS, and CS/BNC vs. temperature in nitrogen atmosphere. The BNC started to degrade from 203°C and achieved the maximum decomposition rate at 258.9°C as indicated by derivative thermogravimetric curve (DTG) plotted in Figure 6B. Compared to BNC, CS had a slightly higher starting decomposition temperature of 258°C and the maximum decomposition rate was recorded at 302.0°C. CS/BNC composites displayed only one weight loss curve intermediated between that of pure chitosan and pure BNC samples. The DTG plot shows the peaks of decomposition for pure BNC and CS sharply appeared at 258.9 and 302.0°C, respectively. The broad decomposition rate peaks of CS/BNC composites were the results of superposition of the two materials. The maximum decomposition rates of CS/BNC composites peaked at 283.1, 292.7, and 298.6°C for 0.25%CS/BNC, 0.50%CS/BNC, and 0.75%CS/BNC respectively, showing a rising trend with the addition of CS.

Both the IR and TGA results indicated that CS was well-incorporated with BNC and generated strong interaction with the cellulose molecules.

#### Water holding, absorption, and retention capacity

The water holding, absorption and retention properties of the integral fabric-reinforced composites and the fabric-removed composite hydrogel coats were investigated separately. As shown in the Table 1, the WHC of the hydrogel coat striped from the reinforced hydrogel composites reached 69.1, 75.6, 84.3, 89.1 g/g for BNC, 0.25%CS/BNC, 0.50%CS/BNC, and 0.75%CS/BNC respectively, indicating the improvement in the WHC along with the increase of chitosan concentration in culture media. However, if measured with the reserved fabric skeleton, the WHC of integral fabric-reinforced hydrogel composites decreased considerably to 18.5, 30.0, 33.2, 45.4 g/g for the fabric-reinforced BNC, 0.25%CS/BNC, 0.50%CS/BNC, and 0.75%CS/BNC, respectively.

**Table 1:** **Water holding capacity and absorption behavior of naked cotton gauze, integrated reinforced CS/BNC composites and their separated hydrogel coats only**.

**Sample**	WHC^*^ (g/g)	WAC^*^ (g/g)	(W_1_- W_2_)W_3_^*^(g/g)	(W_2_- W_3_)W_3_^*^(g/g)	(W_1_- W_2_)(W_2_-W_3_)^*^(g/g)
Cotton gauze	Null	5.7±0.3	5.3±0.5	0.4±0.1	11.8±0.6
BNC (coat)	69.1±5.1	56.0±3.8	48.1±3.6	7.8±0.1	6.2±0.5
0.25%CS/BNC (coat)	75.6±4.0	65.1±4.8	57.8±4.0	7.9±0.9	7.3±0.7
0.50%CS/BNC(coat)	84.3±6.4	67.2±3.2	59.0±3.1	8.2±0.3	7.2±0.5
0.75%CS/BNC (coat)	89.1±7.3	78.4±5.2	69.0±6.2	9.5±1.1	7.1±0.5
BNC (integrated)	18.5±1.0	19.7±1.3	16.2±2.0	2.5±0.5	6.9±1.0
0.25%CS/BNC (integrated)	30.0±2.0	29.1±1.9	26.0±2.4	3.1±0.6	8.4±1.5
0.50%CS/BNC (integrated)	33.2±1.6	34.1±1.6	30.2±2.2	3.9±0.2	7.7±0.4
0.75%CS/BNC (integrated)	45.4±2.2	42.7±3.3	38.0±2.8	4.8±0.5	8.0±0.2

* *Each test was repeated eight times, and the means ± standard deviations were given*.

WAC, a parameter reflecting the amount of fluid absorbed by the wound dressing on a basis of gram water per gram mass, is an important feature to absorb wound exudates to maintain a moist physiological and clean environment. The WAC of the freeze-dried hydrogel coat achieved 56.0, 65.1, 67.2, 78.4 g/g for BNC, 0.25%CS/BNC, 0.50%CS/BNC, and 0.75%CS/BNC respectively, which surpassed the naked cotton gauze (5.7 g/g) seven-folds and more. The WAC was improved by *in situ* compositing with CS and improved with increase of CS addition, which is in accordance with a previous report using static cultures (Phisalaphong and Jatupaiboon, 2008). After coating the fabric with nano-fibrillar BNC hydrogel, the overall WAC of the fabric-reinforced BNC and -reinforced iCS/BNC (*i* = 0.25, 0.50, 0.75%) was improved to a decent level of 19.7, 29.1, 34.1, and 42.7 g/g, respectively, which is remarkably bigger than 5.7 g/g of the cotton gauze fabric. The BNC- and CS/BNC-coating fabric composites should be considered as promising dressing materials.

In order to investigate the fluid distribution of CS/BNC composites, a centrifugation method in British Pharmacopeia for alginate wound dressings was performed (Qin, 2004, 2008). The absorbed fluid in dressings can be divided into two kinds: the fluid held between the fibers (W_1_ − W_2_) and liquid held inside the fibers (W_2_ − W_3_). The former is more inclined to migrate along textile structure, which could cause macerations of surrounding healthy skin in clinical application, while the later is more stable, which is helpful to maintain an ideal moist healing environment (Qin, 2004, 2008). The ratio of (W_1_ − W_2_)/(W_2_ − W_3_) reflects the fluid distribution within wound dressings.

**Figure 7:**
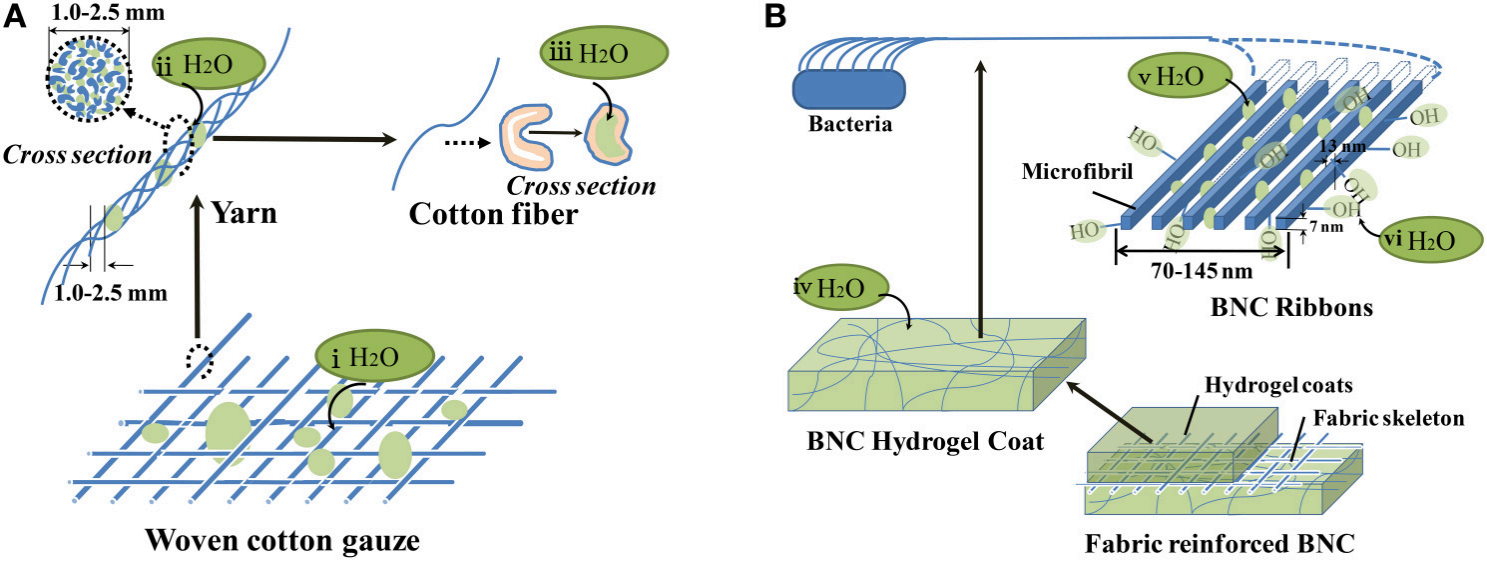
**Hypothesized absorption distribution in the woven cotton gauze (A) and BNC hydrogel (B): water holding in the meshes of warp and weft (i); water retained in crevices of yarns (ii); water absorbed into cotton fibers (iii); water retained in the nano-fibrillar networks (iv); water interred BNC nano-fiber (v); water bounded to BNC nano-ribbons (vi)**.

Cotton gauze has a hierarchically structural water distribution in the single cotton fiber, multiple yarns, and woven or knitted structure, with a decreasing water mobility (as shown in Figure 7A; Marsh et al., 1953; Qin, 2004). By centrifuging, the spun-out fluid (W_1_ − W_2_, i.e., the fluid held between the fibers) is assigned to those held by meshes of woven or knitted structure (Figure 7Ai) and by interstices in multiple yarns (Figure 7Aii), while those immovable liquid (W_2_ − W_3_, i.e., the liquid held inside fibers) is supposed to the fluid absorbed into the cell wall of single fiber (Figure 7Aiii). Table 1 shows that the bulk of liquid absorbed into cotton gauze by capillary forces was 5.3 g/g as an index of (W_1_ − W_2_)/W_3_ and the ratio of (W_1_ − W_2_)/(W_2_ − W_3_) was 11.8 g/g. In contrast, BNC has a porous nano-fibrillar network constituted by cellulose ribbons of sub-100 nm, therefore the fluid can be held in the nano-porous structure by capillary (Figure 7Biv) and held in the nanofibers (Figure 7Bv) or firmly bound to the plentiful hydroxyl of BNC nanofiber surface (Figure 7Bvi; Klemm et al., 2005). Benefited from the supramolecular structure with high porosity, BNC can absorb abundant liquid within its nanofibril network as the index of (W_1_ − W_2_)/W_3_ reached 48.1 g/g. And the water absorption can be assigned to difficult-to-migrate reaching 7.8 g/g, which is a magnitude significantly greater than the cotton gauze (0.4 g/g) (Table 1). The ratio of (W_1_ − W_2_)/(W_2_ − W_3_) of BNC was detected to 6.2 g/g, indicating water retention ability of BNC is better than cotton gauze and this performance is close to some commercial alginate dressings, for instance, a Curasorb™ dressing had 32.0 g/g of (W_1_ − W_2_)/W_3_ and 8.62 g/g of (W_1_ − W_2_)/(W_2_ − W_3_) (Qin, 2004). After compositing with CS, both (W_1_ − W_2_)/W_3_ and (W_2_ − W_3_)/W_3_ of the BNC hydrogel coat were improved, indicating the hydrophilic CS polymers improve both the structural water absorption capacity and the ability to bound water. The ratios of (W_1_ − W_2_)/(W_2_ − W_3_) of CS/BNC composites were 7.3, 7.2, and 7.1 g/g for 0.25%CS/BNC, 0.50%CS/BNC, and 0.75%CS/BNC respectively, which are slightly higher than 6.2 g/g of BNC. As a whole material, Table 1 shows the ratio of (W_1_ − W_2_)/(W_2_ − W_3_) for fabric-reinforced BNC and iCS/BNC (*i* = 0.25, 0.50, 0.75%) were 6.9, 8.4, 7.7, 8.0 g/g respectively, which indicates the overall liquid retention behaviors of the fabric-reinforced BNC and CS/BNC composites are near to the corresponding hydrogel coats. This is because the hydrogel coats performed the majority of the absorption.

#### Mechanical properties

**Figure 8:**
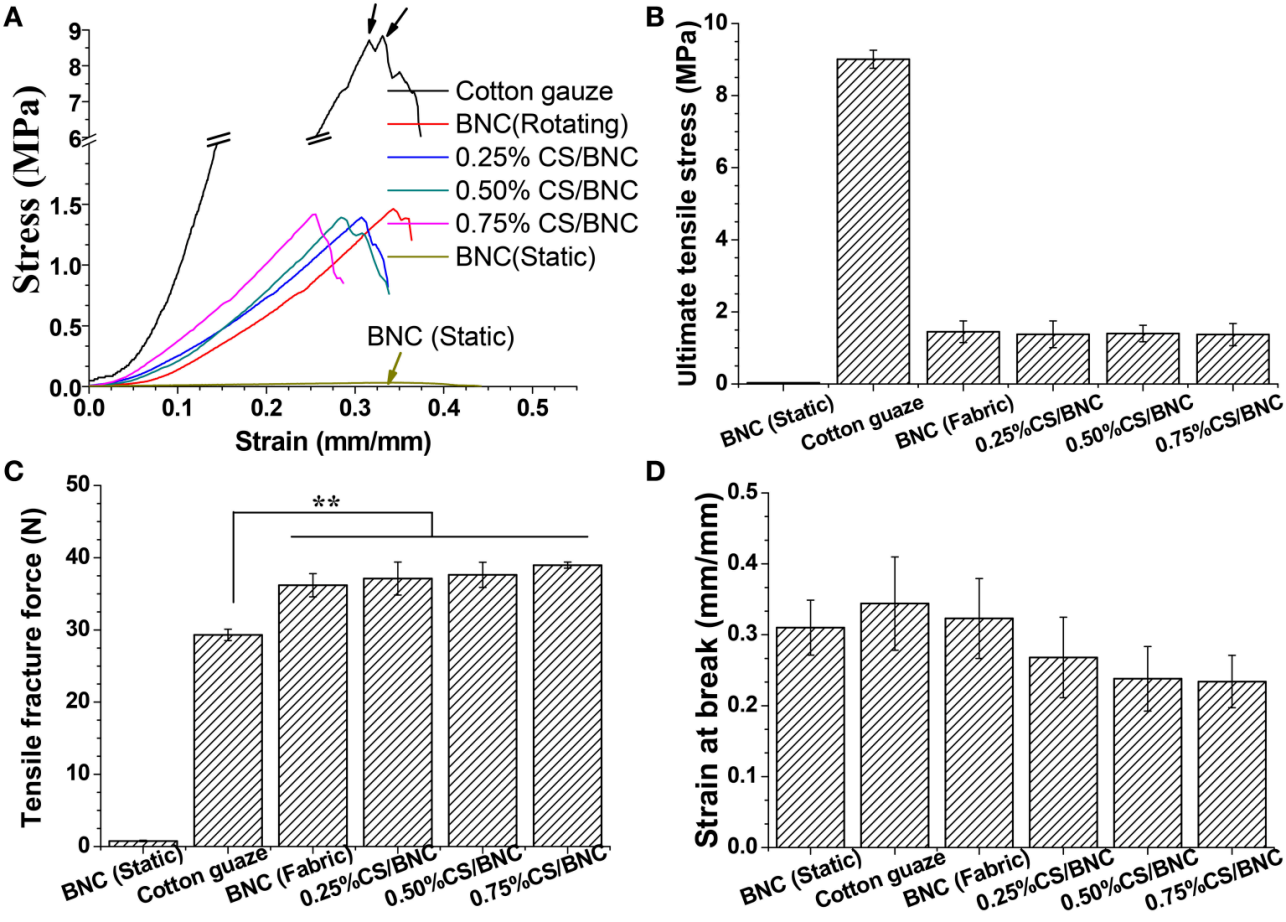
**Tensile properties of the pure BNC sheet (static), naked cotton gauze, fabric-reinforced BNC (rotating), and fabric-reinforced CS/BNC composites: (A) representative strain–stress curves, (B) ultimate tensile stress, (C) tensile fracture force, (D) strain at break**. The tests were performed in octuplicate, and means ± standard deviations were given.

The tensile mechanical properties of the fabric-reinforced BNC and fabric-reinforced CS/BNC composites are shown in Figure 8 in comparison with the uncoated cotton gauze and pristine BNC sheets from a 10-d static culture. The ultimate tensile stress of the high water swollen pristine BNC hydrogel sheets from static culture was only 0.03 ± 0.04 MPa, which is apparently far weaker than ordinary fabrics (in this case cotton gauze showed 9.0 ± 0.3 MPa, Figure 8B). The ultimate tensile stress of the fabric-reinforced BNC and -reinforced CS/BNC composites ranged from 1.4 to 1.5 MPa (Figure 8B) and no significant difference was found among them. These relatively low values should be attributed to the remarkable thickness of the fabric-reinforced BNC and CS/BNC composites since the tensile stress (MPa) is a normalized value from tensile force (N) through dividing by the cross-sectional area (m^2^) of the material. Figure 8C shows that the fabric skeletons strengthen the hydrogel obviously, resulting in immediately boosting in tensile fracture strength. Not surprisingly, with similar hydrogel thickness, the fabric-reinforced BNC and iCS/BNC (*i* = 0.25, 0.50, 0.75%) obtained similar ultimate tensile stress values. However, the tensile fracture force of reinforced BNC hydrogel or reinforced CS/BNC composites all surpassed 36 N (Figure 8C), which was significantly higher than the uncoated cotton gauze (29.3 N). The results imply a composite effect that the BNC or CS/BNC hydrogel coat could reinforce the fabric skeleton as well. Noteworthily, the increments in fracture force values were still far higher than the pristine BNC hydrogel sheets without fabric skeleton (0.8 N). This indicates the increments in facture force should be mainly attributed to the cohesion between the hydrogel coats and the fabrics rather than the hydrogel coats themselves. BNC nano-filaments attachment on the surface of natural hemp fibers was found to greatly improve the interfacial shear strength of fibers, which creates strong viscous friction on fiber surface (Pommet et al., 2008). Therefore, the BNC attachments would enhance the interfacial adhesion among cotton fibers in each individual yarns, as well as enhance the interfacial adhesion between warp and weft yarns, by which the cotton gauzes got more integrated and reinforced. The cohesive effects could decrease the interfacial displacement in inter- or inner- yarns, for which the values of strain at break of fabric-reinforced BNC (0.32 mm/mm) and -reinforced CS/BNCs (0.23 to 0.27 mm/mm) were slightly lower than the uncoated cotton gauze (0.34 mm/mm, as shown in Figure 8D). The cotton gauze fractured in the form that yarns broke one by one, resulting in the multiple peaks in the stress-strain curve as indicated by black arrows in Figure 8A. But after coating with BNC or CS/BNC hydrogel, it fractured almost simultaneously at all stretched yarns (reflected by the single-peaked stress-strain curves in Figure 8A). It also indicates an improvement in the integration between yarns. The results suggest that the inner frame of fabric can be introduced into BNC hydrogel to augment its tensile property, and the fabric itself also can be reinforced by BNC coating for more intense bonding strength in inter- and inner-yarns.

#### Antibacterial ability

**Figure 9:**
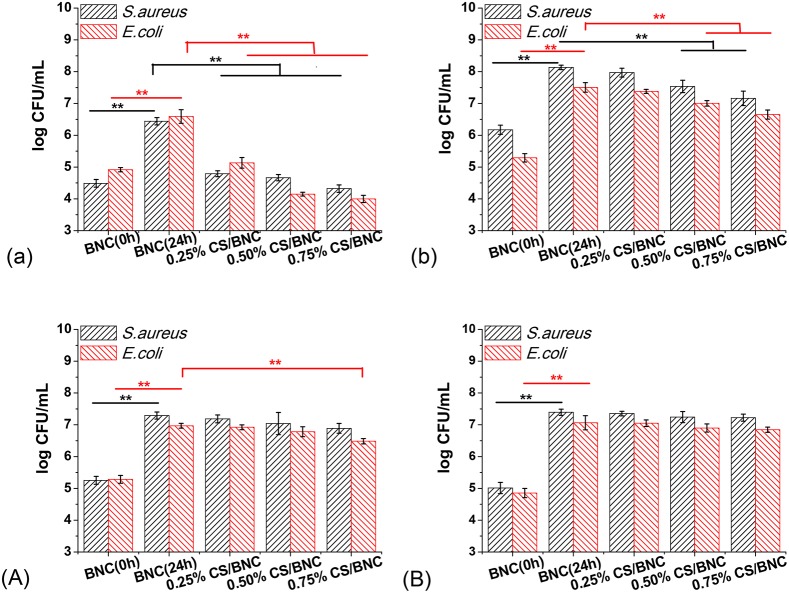
**Antibacterial activity of the fabric-reinforced CS/BNC composites in two different states against. ***S. aureus*** and ***E. coli*** using two different methods: (a) lyophilized samples and (b) hydrogel samples were evaluated with the absorption method in 24 h touching, and (A) lyophilized samples and (B) hydrogel samples were evaluated with the shake-flask method in 24 h shaking incubation**. The experiments were performed in triplicate, and the logarithmic mean values of bacterial colonies and standard deviations were given.

Antibacterial ability of the reinforced-CS/BNC in both hydrogel state and lyophilized state was investigated by using two methods: absorption method and shake-flask method (saline). The results of antimicrobial assessment are shown in Figure 9 and Table 2.

**Table 2:** **The percentage growth reduction (R) and antibacterial activity (A) of antibacterial tests for fabric-reinforced composites**.

**State**	**Samples**	**Absorption method**				**Shake-flask method**			
		***S. aureus***		***E. coli***		***S. aureus***		***E. coli***	
		**R (%)**	A^*^	**R (%)**	A^*^	**R (%)**	A^*^	**R (%)**	A^*^
Lyophilized	0.25%CS/BNC	97.79	1.65	96.67	1.46	16.07	0.08	1.32	0.05
	0.50%CS/BNC	98.34	1.77	99.67	2.44	27.36	0.22	32.62	0.19
	0.75%CS/BNC	99.24	2.12	99.76	2.59	56.97	0.38	67.24	0.49
Hydrogel	0.25%CS/BNC	29.71	0.16	27.36	0.12	9.89	0.04	10.35	0.01
	0.50%CS/BNC	73.22	0.60	69.26	0.50	26.21	0.15	36.13	0.16
	0.75%CS/BNC	88.37	0.96	85.98	0.85	32.05	0.17	44.53	0.22

* *The growth value on the control sample (F-value) for all the tests was more than 1.5, which verifies the effectiveness of antibacterial tests*.

By using the absorption method (Figure 2A), the bacterial inocula directly touched with control and test samples. For the lyophilized samples, the number of bacterial colonies obtained from all the CS/BNC composites was significantly lower than the BNC control group after 24 h contact (Figure 9a). The bacterial reduction percentage ranged from 97.79 to 99.24% with a corresponding *A*-value ranging from 1.65 to 2.12 for *S. aureus*, and from 96.67 to 99.76% with a corresponding *A*-value ranging from 1.46 to 2.59 for *E. coli*, respectively (Table 2), demonstrating a positive broad-spectrum antibacterial effect. In contrast with the BNC (24 h), the bacterial growth on CS/BNC composites was significantly lower (decreased more than 1 logarithmic value, as shown in Figure 9a), showing a remarkable inhibition on bacterial growth (bacteriostatic activity). However, as compared with the bacterial growth after just inoculation (BNC 0 h), the bacterial growth on CS/BNC composites after 24 h contact only had a small reduction (less than 1 logarithmic value), even though slightly increased for 0.25% CS/BNC, exhibiting almost no bactericidal ability. This result suggests that the lyophilized CS/BNC composites mainly exerted bacteriostatic effect (inhibition function on growth) instead of bactericidal effect. This result is in accordance with previous literatures reporting that the antimicrobial activity of CS is bacteriostatic rather than bactericidal (Raafat et al., 2008; Kong et al., 2010). For the gelatinous samples (Figure 9b), the bacterial numbers on all CS/BNC composites were lower than the BNC group at 24 h, but higher than the number after just inoculation (BNC 0 h), also showing a bacteriostatic ability. The antibacterial effect of hydrogel samples of 0.25%CS/BNC, 0.50%CS/BNC, and 0.75%CS/BNC increased with the addition of CS and the bacterial reduction percentage was 29.71, 73.22, 88.37% against *S. aureus*, and 27.36, 69.26, 85.98% against *E. coli*, respectively (Table 2). Taking into account the above results it is suggested that the antibacterial effect of lyophilized samples was much stronger than that of the corresponding hydrogel samples.

A modified shake-flask method (Singh et al., 2012) was also applied (Figure 2B) on the basis of the standard dynamic shake-flask method (ASTM-E2149, 2010), where PBS was used instead of nutrient broth and the number of bacterial colonies were determined by a plate counting method. The results are shown in Figures 9A,B. Without nutrient addition, the F value showed a significant bacterial growth corresponding to 2.01 and 1.68 with the freeze-dried control samples, and showed a significant bacterial growth corresponding to 2.38 and 2.21 with the hydrogel control samples against *S. aureus* and *E. coli*, respectively (Table 2). Figures 9A,B show the number of viable bacterial cells in the CS/BNC-added buffer solution was slightly decreased as compared to the bacterial population of BNC (24 h) group. However, both the lyophilized samples and the gelatinous samples exhibited very weak bacteriostatic effect in the dynamic touch because the percentage of microbial reduction for majority test groups was less than 50% against both *S. aureus* and *E. coli* (as shown in Table 2). However, the lyophilized 0.75%CS/BNC showed a relatively significant reduction against *S. aureus* and *E. coli* in viable bacterial counts in comparison to BNC (24 h), corresponding to a percentage growth reduction of 56.97 and 67.24%, respectively (as shown in Table 2).

In addition to the two quantitative tests, a semi-quantitative test with the agar plate diffusion method was also implemented to evaluate both hydrogel and lyophilized samples. However, no visible inhibition zones have been observed in all the CS/BNC samples for both bacterial species.

The results showed the antibacterial activity of reinforced CS/BNC composites had great discrepancies among the different evaluation methods and also among different sample's physical states. Antibacterial activity was more remarkable in lyophilized state rather than hydrogel state, and was effectively detected in absorption method rather than in shake-flask method. This is because the antibacterial effect of CS is dose-dependent (Raafat et al., 2008), and is also heavily influenced by physical states and solid shapes (Kong et al., 2010). Although the CS concentration in culture media was very low (0.25 to 0.75%, w/v) during *in situ* bio-synthesis of CS/BNC composites, CS proportion was able to reach as high as approximate 40% in the dehydrated CS/BNC matrix. This makes internal space of CS/BNC matrix be of regionally high CS content, which forms a strong bacteriostatic micro-environment. The bacterial growth was strongly inhibited by long-time exposure to the local CS-rich matrix in the absorption method. However, in the shake-flask method, the regionally CS-rich CS/BNC matrix could not affect the bacterial growth of surrounding bacterial suspension in overall only by means of the repeatedly and instantly contacting with the CS/BNC of a standard addition amount (0.01 g/mL). The results showed that the bacteriostatic effect of CS/BNC composites was confined within the matrix only, instead of spreading around. The lyophilized sponge-like composites are able to rapidly absorb the water of the droplets of liquid bacterial inocula into internal matrix in the absorption method, while the liquid inocula should only be spread on the superficial surface of hydrogel samples after dropping since the hydrogel samples are saturated with water. The water absorption into the lyophilized samples makes more bacterial cells exposed to or contacted with the CS/BNC matrix, resulting in a better bacteriostatic effect than the gelatinous samples. From another point of view, the practical CS proportion in the gelatinous samples was much lower than that in the lyophilized samples by taking into account water proportion, which should lead to more survival cells. In agar diffusion tests, no inhibition zone was apparent for all the samples, reflecting the bacteriostatic effect of CS/BNC is only confined within the matrix.

## Conclusions

Fabric-reinforced CS/BNC composite sheets were prepared successfully through the *in situ* rotating culture technology, and the inherent structural characteristics of BNC hydrogel coat were preserved. The fabric skeleton sustained most of the tensile force, which directly boosts the tensile strength. The fabrics functioned as the supports of cellulosic biofilm immobilization in rotating cultures. This rotating culture process not only has higher productivity than static culture, but also reduces the inhibition effects of the CS on *G. xylinus* growth and cellulose production. CS dispersed in culture media could be incorporated into BNC matrix successfully with this rotating culture technology, and could be retained in the hydrogel substrate after the rigorous alkaline purification process. Addition of CS into the culture media modified the nano-fibrillar networks of BNC and improved valuable features including water absorbing and maintaining properties. The CS/BNC composite sheets showed a good bacteriostatic effect, particularly in the lyophilized sponge state. These features endow the fabric-reinforced CS/BNC composites with great potential as excellent medical materials for wound dressings. Furthermore, the proposed *in situ* rotating culture technology could be used to integrate other materials including particles and soluble polymers into the nanofibril network of BNC to generate new composites.

## Author contributions

FFH designed and coordinated the study and revised the manuscript. PZ and LC contributed the preparation of the manuscript. PZ carried out most of the experiments and analyzed the results. QZ performed some antibacterial tests. All authors read and approved the final manuscript.

### Conflict of interest statement

The authors declare that the research was conducted in the absence of any commercial or financial relationships that could be construed as a potential conflict of interest. FFH is an author on patent applications on the construction of bioreactor and the *in situ* dynamic culture technology for biofabrication of chitosan/BNC composites.
